# The mediating role of self-efficacy in the relationship between health literacy, health status and psychological well-being among German trainees and students in nursing

**DOI:** 10.3389/fpubh.2024.1436665

**Published:** 2024-12-18

**Authors:** Ramona Otto, Ivonne-Nadine Jürgensen, Albert Nienhaus, Peter Koch

**Affiliations:** ^1^Competence Center for Epidemiology and Health Services Research for Healthcare Professionals (CVcare), Institute for Health Services Research in Dermatology and Nursing (IVDP), University Medical Center Hamburg-Eppendorf, Hamburg, Germany; ^2^Department of Nursing and Management, Faculty of Business and Social Science, University of Applied Sciences Hamburg, Hamburg, Germany; ^3^Department for Occupational Medicine, Hazardous Substances and Health Sciences (AGG), German Social Accident Insurance for the Health and Welfare Services (BGW), Hamburg, Germany

**Keywords:** health literacy, self-efficacy, nursing, vocational education, students, health status, psychological well-being, health care workers

## Abstract

**Introduction:**

Health literacy is considered a determinant of several health-related behaviors and outcomes. Health literacy can be seen as a particularly relevant resource for health maintenance and promotion for young adults at the beginning of their challenging vocational training in nursing. However, the underlying mechanisms and the roles of other influencing factors such as self-efficacy remain unclear and need to be understood to successfully tailor interventions.

**Methods:**

This cross-sectional study aims to examine the relationships between health literacy, self-efficacy, health behavior and health status among 556 German trainees and students in nursing. Data were collected using paper-pencil and online questionnaires between January and June 2021. Mediation models were assessed, consisting of the direct pathways between health literacy and health status; health literacy and health behavior; and indirect pathways via self-efficacy.

**Results:**

The findings showed that health literacy positively affected self-rated health status (*ß* = 0.05, *t* = 4.35, *p* < 0.001) and psychological well-being (*ß* = 0.28, *t* = 3.34, *p* < 0.001). The effect of health literacy on psychological well-being was fully mediated through self-efficacy (*Z* = 265 4.91, *p* < 0.001) while the effect of health literacy on self-rated health status was partially mediated through self-efficacy (*Z* = 5.06, *p* < 0.001). In this cohort, there was no significant association between either health literacy or self-efficacy and health behavior.

**Conclusion:**

Self-efficacy should be taken into account when investigating health literacy and its possible relationships with health-related outcomes. Furthermore, it should be considered as an objective for health literacy interventions as well as health promotion measures for nursing students and trainees.

## 1 Introduction

At the end of 2021, there were more than 56 thousand people in Germany undergoing educational training in nursing (1). The majority of these trainees are between the ages of 18 and 25 (1). This phase of emerging adulthood (2) is known to be challenging, as it involves the major life transition of graduating from school and entering the job market as well as personal maturation, taking on additional responsibility and potentially moving out of the parental home.

Additionally, for these trainees, vocational education and working as a nurse in general involves high demands and stresses. The COVID-19 pandemic has further deteriorated working conditions, leaving nurses with a higher likelihood of developing post-traumatic stress disorder (PTSD), anxiety, stress and burnout (3). German health insurance data showed that nurses’ annual sick-leave days are significantly higher compared to the rest of the workforce, with musculoskeletal disorders and mental health issues being the leading causes (4, 5). These figures emphasize the need for both mental and physical health promotion for this target group.

With a shortage of nearly 6 million nurses worldwide (6), promoting a healthy nursing workforce has a particularly important role to play in terms of decreasing job turnover and increasing job attractiveness. Strengthening health literacy is one way to improve and maintain physical and psychological well-being, which has been demanded in the National Health Literacy Action Plan for Germany (7). Health literacy is a concept that has increasingly been addressed in public health research (8, 9). A number of models and definitions have been published on health literacy. Based on the literature Sörensen et al. described it as an individual’s competencies in accessing, understanding, appraising and applying health-related information (8). Health literacy is determined by several demographic factors such as gender, social and financial status, migration background, education, age and the presence of chronic diseases (10–12).

Studies have underlined associations between higher levels of health literacy and better health outcomes, such as higher psychological well-being (13, 14). Associations have also been identified between health literacy and both self-rated health (15, 16) and health behavior (15). Low levels of health literacy have been associated with lower use of preventive health care services (17). A Europe-wide study found that 48% of the respondents reported limited health literacy. The study observed a significant disparity in terms of the percentage of respondents reporting limited health literacy between countries, from just 29% in the Netherlands to 62% in Bulgaria. For Germany, the researchers found limited health literacy in 46% of the study population (10). However, according to a more recent representative study, the share of people with limited health literacy in Germany is much higher, at almost 60% (11). Within the group of nursing trainees, health literacy levels were found to be sufficient for around half of the cohort at the beginning of their vocational training (18).

In addition to its health-promoting aspects, an adequate level of health literacy might help nursing trainees and students to provide efficient health care after their graduation. The COVID-19 pandemic and the far-reaching associated changes and insecurities in health care provision have made it abundantly clear how important sufficient health literacy is for health professionals. Aligning an individual’s skills with the demands and complexities of their environment is one important goal for health literacy (19). Furthermore, health literacy is regarded as a facilitator in terms of the participation and empowerment of individuals (20), which could potentially lead to an improvement of conditions for both nurses and patients in the long run.

When looking into health literacy, it is important to consider self-efficacy as an influential factor. Self-efficacy has repeatedly been associated with health literacy (21–25). It is defined as someone’s perceived ability to successfully execute behaviors to reach a certain outcome (26). Its role in the mechanism of the effect of health literacy on health-related outcomes has been previously investigated in other populations, where it influenced the relationship between health literacy and health outcomes (21, 27, 28). However, the findings in this area have been inconsistent and the underlying mechanisms remain unclear. In addition, most studies in this field target older population groups with certain medical conditions rather than younger and healthier target groups. Hence, there is a lack of evidence providing information on how to target health literacy improvements specifically in nursing trainees and students, taking also self-efficacy into account.

This study sought to investigate whether self-efficacy mediates the effect of health literacy on health behavior and health status in trainees and students in nursing in Germany. In contributing to the understanding of how health literacy and self-efficacy affect health behavior and health outcomes in a young population, vocational schools and universities could be encouraged to tailor interventions to improve health literacy and self-efficacy among their students.

## 2 Methods

### 2.1 Sample and data collection

The cross-sectional study was performed in the federal states of Hamburg, Lower Saxony, Schleswig-Holstein, Mecklenburg-Western Pomerania and Berlin in Northern Germany between January and July 2021. Trainees were recruited in vocational schools offering generalized nursing education. Eligible vocational schools were identified by online research and contacted regarding their willingness to participate in the study. Of the 92 eligible vocational schools, 66 agreed to participate in the study (response rate 72%). Using a paper-pencil approach, a total of 2,641 trainees in their first few weeks of vocational training were invited to participate in the study. Questionnaires were anonymized by an independent center. Nursing students were recruited at the Hamburg University of Applied Sciences (Nursing & Management Department). 37 students were approached during lectures and asked to fill in an online questionnaire. The manner in which questionnaires were disseminated differed between trainees and students. Students were approached by the researchers at university lectures and asked to fill in the online questionnaire onsite. Students were given time to complete the questionnaire during the lecture and the researchers were present to answer questions. For trainees, cooperating vocational schools disseminated the paper-pencil questionnaires. As a result, the exact conditions of the recruitment process remain unclear to the researchers and might differ between vocational schools. Since the number of participating vocational schools was high and they were spread across northern Germany, the recruitment process could not be supervised and executed as close as it was the case for the participating University.

The purpose of collecting data from nursing trainees and students was to evaluate possible long-term differences in health literacy due to the higher degree of professionalization on the academic educational pathway. These analyses will be performed using data collected one year later (t1).

There were 556 questionnaires eligible to be included in this analysis, 528 of which were completed by trainees (response rate 20%) and 28 by students (response rate 76%).

### 2.2 Ethical considerations

An ethics endorsement was obtained from the Hamburg Medical Association (PV5670). Furthermore, the study was approved by the school authorities in the federal states of Berlin, Lower Saxony, Schleswig-Holstein and Mecklenburg Lower-Saxony. The school authority in the federal state of Hamburg did not approve the study. Study participants were provided with details about the purpose of this study, the fact that participation was voluntary, their right to refuse to answer or withdraw from the study, and the risks and benefits of participation. All participants signed the declaration of consent.

### 2.3 Measures

#### 2.3.1 Demographic information

The questionnaire incorporated several questions on sociodemographic data, covering age, gender, nationality, country of birth and highest level of education. After reviewing the literature, age, gender and education level were included as control variables in the mediation analyses.

#### 2.3.2 Health literacy

The short form of the German Health Literacy Survey Questionnaire (HLS-EU-Q16) (29, 30) was used to assess the self-reported health literacy of the cohort. The instrument consists of 16 items scored on a 4-point Likert scale ranging from 0 (very difficult) to 4 (very easy). The instrument touches on three main areas of health literacy: health care, disease prevention and health promotion. Following the recommended procedure, each Likert scale was dichotomized and simple sum scores were calculated to provide a score ranging from 0 to 16. Higher scores indicate better health literacy. Based on the sum score, three health literacy levels can be derived: sufficient (13–16 points), problematic (9–12 points) and inadequate (0–8 points). Only participants with at least 14 valid answers were included in further analyses (30). The psychometric properties of the HLS-EU-Q16 are acceptable and its sum score shows a high correlation with the score of the long version (30). Cronbach’s alpha coefficient for the HLS-EU-Q16 was 0.83 in the present study, indicating good internal consistency.

#### 2.3.3 Self-efficacy

Self-efficacy was measured using the General Self-Efficacy Scale (GSE) (31) developed by Schwarzer & Jerusalem. The 10 items of the scale are rated using a 4-point Likert scale ranging from 1 (not at all true) to 4 (exactly true). Scores are summed up to provide a score ranging from 10 to 40. Higher scores indicate more self-efficacy. Cronbach’s alpha coefficient for the GSE was 0.82 in the present study, indicating good internal consistency.

#### 2.3.4 Health status

Four indicators of health status were measured:

*Self-rated health status* was assessed subjectively using a 5-point Likert scale (1 = excellent, 2 = very good, 3 = good, 4 = less good, 5 = poor) (32). Categories were dichotomized between “good” (excellent/very good/good) and “poor” (less good/poor).

*Medical diagnoses* within the past 12 months were assessed using a short version of the Work Ability Index (33). Categories were built based on the number of medically diagnosed conditions (None/1–2 diseases/3–4 diseases/5 or more diseases).

*Psychological well-being* over the previous 2 weeks was measured using the German version of the 5-item World Health Organization Well-Being Index (WHO-5) (34). The instrument contains 5 questions with response options ranging from 0 (at no time) to 5 (all of the time). Item scores were summed up to provide a total score between 0 and 25, with higher scores indicating better psychological well-being. The index was dichotomized using the cutoff value suggested in the literature; consequently, scores under 13 points were categorized as poor psychological well-being. Cronbach’s alpha coefficient for the WHO-5 was 0.86 in the present study, indicating good internal consistency.

*Body Mass Index (BMI)* was calculated based on self-reported body height and weight. BMI was categorized as follows: underweight (BMI < 18.5), normal weight (BMI between 18.5 and under 25), overweight (BMI between 25 and under 30) and obese (BMI > 30) (35).

#### 2.3.5 Health behavior

Four indicators measuring health behavior were measured as follows:

*Smoking behavior* was measured by one item, asking whether participants were currently smoking. The possible responses were “Yes, daily,” “Yes, occasionally,” “No, I have smoked in the past” and “No, I have never smoked.” Responses were summarized as “yes” (daily or occasionally) and “no” (non-smoker or ex-smoker) (36).

*Alcohol consumption* was assessed using the AUDIT-C questionnaire (37), consisting of three questions. Item scores were summed up to provide a total score ranging from 0 to 12 points, with higher scores indicating higher levels of alcohol consumption. The index was dichotomized using different cutoff values for risky alcohol consumption for men and women (men: > 4 points; women: >3 points).

*Physical activity* was assessed by one question capturing how frequently each participant is physically active. The possible responses were “no physical activity,” “less than 1 h per week,” “regularly, 1–2 h per week,” “regularly, 2–4 h per week” and “regularly, more than 4 h per week” (38). Responses were dichotomized between “sufficient” (2–4 h per week or > 4 h per week) and “insufficient” (1- < 2 h per week, < 1 h per week or none) activity levels.

*Eating behavior* was measured using self-reports based on a food frequency list including 15 food categories (39). Responses were analyzed in accordance with the authors’ framework to provide a score ranging from 0 to 30. Based on their score, participants were assigned to the following categories: optimal nutritional pattern (16–30 points), normal nutritional pattern (13–15 points) and unfavorable nutritional pattern (0–12 points). A binary variable was computed by summarizing optimal and normal nutritional patterns into one category. Fast food consumption was analyzed separately and dichotomized between “≤ once/week” and “> once/week.”

The indicators of health behavior described above were used to calculate an overall score, ranging from 0 (unfavorable health behavior) to 5 (favorable health behavior).

### 2.4 Data analyses

To gain a deeper understanding of the cohort, descriptive statistics were used for all relevant items. Associations were explored using analysis of variance (ANOVA) for continuous outcome variables, and Pearson’s chi-squared test for categorical data. Mediation analyses were performed using the PROCESS macro Version 4.1 for SPSS developed by Hayes (40) to test the hypothesized mediating role of self-efficacy in the relationship between health literacy and health status. A Sobel test was performed to determine whether indirect effects were of statistical significance. All statistical analyses were performed using IBM SPSS for Windows Version 27. The criteria for mediation analyses were based on Baron and Kenny (41). Results with a *p*-value under the threshold of 0.05 were considered statistically significant. Missing data was handled using pairwise deletion. Gender-diverse participants (*N* = 3) were randomly assigned to one of the binary gender groups for the purpose of the analyses as the gender-diverse group was too small to be analyzed separately.

## 3 Results

### 3.1 Characteristics of the sample

The majority of the 556 participants were women (*N* = 427, 76.8%). The age range was 17–57 years with a mean of 24.6 years (SD = 8.1). Three participants identified as gender-diverse and were randomly assigned to one of the binary gender groups for the analyses. Most participants were of German nationality (*N* = 408, 77.3%) and had graduated the higher secondary school (*N* = 250, 45.5%). Trainees had a higher mean age than students (24.7 vs. 23.3), but this difference was not statistically significant (*p* = 0.313). Women made up a higher proportion of trainees than students (77.5% vs. 71.4%), but this difference in gender distribution was not statistically significant (*p* = 0.46). Table 1 provides additional information about the participants.

**Table 1:** Demographic characteristics of the study cohort.

Demographic variable	Category	Female (*N* = 429)	Male (*N* = 127)	Total (*N* = 556)	*p*
Age	X (SD)	24.7 (8.3)	24.5 (7.3)	24.6 (8.1)	0.782^1^
	Range	17-57	17-57	17–57	
Nationality	German	320 (78%)	88 (74%)	408 (77%)	0.326^2^
	Other	89 (22%)	31 (26%)	120 (23%)	
	n missing	20	8	28	
School education	Lower secondary school (Hauptschule)	42 (10%)	13 (10%)	55 (10%)	0.731^2^
	Higher secondary school (Realschule)	195 (46%)	55 (44%)	250 (46%)	
	Vocational training college (Fachhochschule)	48 (11%)	13 (10%)	61 (11%)	
	A-Levels (Abitur)	131 (31%)	40 (32%)	171 (31%)	
	Other	8 (2%)	5 (4%)	13 (2%)	
	n missing	5	1	6	
Federal state	Hamburg	20 (5%)	8 (6%)	28 (5%)	0.908^2^
	Lower Saxony	265 (62%)	77 (61%)	342 (62%)	
	Schleswig-Holstein	46 (11%)	15 (12%)	61 (11%)	
	Mecklenburg-Western Pomerania	38 (9%)	9 (7%)	47 (9%)	
	Berlin	60 (14%)	18 (14%)	78 (14%)	
Education path	Trainee	409 (95%)	119 (94%)	528 (95%)	0.459^2^
	Student	20 (5%)	8 (6%)	28 (5%)	

1 ANOVA.

2 Pearson’s chi-squared test.

### 3.2 Health literacy

Table 2 shows health literacy scores for different demographic traits. In the present study cohort, average health literacy scores were rather high (*x* = 12.4, SD = 2.8). Limited health literacy was observed in 44.8% of the cohort, with 35.8% showing problematic and 9.0% inadequate health literacy. Men had significantly higher levels of health literacy than women did (*p* = 0.023). Participants of German nationality were found to have significantly higher scores compared to participants of other nationalities (*p* < 0.01). Participants’ education levels also predicted health literacy levels (*p* = 0.001); however, levels did not increase consistently with higher education levels. There were no statistically significant differences in health literacy scores between trainees and students (*p* = 0.707), different age groups (*p* = 0.161) or federal states (*p* = 0.149).

**Table 2:** Health literacy and self-efficacy scores by demographic traits.

Demographic variable	Category (n)	HL score x (SD)	*p* ^1^	SE score x (SD)	*p* ^1^
Gender	Female (429)	12.3 (2.7)	0.023	28.7 (4.4)	0.022
	Male (127)	12.9 (2.8)		29.8 (4.6)	
Age group	17–18 (76)	13.0 (2.3)	0.161	28.4 (4.0)	0.005
	19–20 (157)	12.5 (2.6)		28.3 (4.5)	
	21–25 (160)	12.1 (3.1)		28.8 (4.3)	
	26–30 (65)	12.5 (2.6)		29.7 (4.3)	
	>30 (98)	12.4 (2.9)		30.2 (4.7)	
Nationality	German (408)	12.8 (2.6)	<0.001	29.0 (4.4)	0.367
	Other (120)	11.4 (3.1)		29.4 (4.5)	
School education	Lower secondary school (Hauptschule) (55)	12.0 (3.0)	0.001	28.2 (4.9)	0.037
	Higher secondary school (Realschule) (250)	12.8 (2.8)		28.9 (4.4)	
	Vocational training college (Fachhochschule) (61)	13.0 (2.3)		29.8 (3.8)	
	A-Levels (Abitur) (171)	11.8 (2.7)		28.8 (4.5)	
	Other (13)	12.9 (2.9)		31.9 (5.2)	
Education path	Trainee (528)	12.5 (2.8)	0.707	29.0 (4.4)	0.065
	Student (28)	12.3 (2.8)		27.5 (4.6)	
Federal state	Lower Saxony (342)	12.6 (2.7)	0.149	29.1 (4.3)	0.193
	Berlin (78)	12.3 (2.7)		29.1 (4.5)	
	Schleswig-Holstein (61)	11.7 (3.3)		28.2 (4.3)	
	Mecklenburg-Western Pomerania (47)	12.9 (2.6)		29.4 (5.2)	
	Hamburg (28)	12.3 (2.8)		27.5 (4.6)	

1 ANOVA.

### 3.3 Self-efficacy

The mean self-efficacy score was 28.97 (SD = 4.43) for the whole sample. Men reported significantly higher levels of self-efficacy compared to women (*p* = 0.022) (Table 2). Higher age groups were significantly associated with higher scores of self-efficacy (*p* = 0.005). Participants’ education levels were significantly associated with self-efficacy scores (*p* = 0.037), but again these did not increase consistently with higher education levels. Trainees had higher self-efficacy scores than students; however, this difference was not statistically significant (*p* = 0.065).

### 3.4 Health behavior and health status

Overall, 69.1% of the cohort exhibited favorable health behavior. However, more than 70% of participants did not achieve a sufficient level of sporting activity, with 21.9% reporting no sporting activity at all. Men reported significantly higher levels of sporting activity (*p* = 0.026). An unfavorable nutrition pattern was found in 41.1% of the cohort, while 29.6% had a normal nutrition pattern and 29.4% had an optimal nutrition pattern. Men’s nutrition patterns were slightly worse than women’s; however, the only statistically significant difference was observed in the area of fast-food consumption (*p* = 0.001). 42.8% of participants reported using cigarettes, with 34.2% reporting daily use. Men smoked significantly more often than women (*p* = 0.001). 30.6% of the cohort reported risky alcohol consumption. Self-rated health status was excellent, very good or good for the vast majority of the cohort (90.6%). Men self-rated their health status significantly better than women (*p* < 0.001). The BMI of the cohort was 25.9 on average, with no significant difference in mean BMI between men and women (*p* = 0.431). Men’s self-reported psychological well-being was significantly better than women’s (*p* = 0.004). Table 3 presents a comprehensive set of health behavior data, broken down by health literacy level.

**Table 3:** Health behavior and health status by health literacy level.

Health literacy level						
Demographic variable	Category	Inadequate (*n* = 50)	Problematic (*n* = 199)	Adequate (*n* = 307)	Total (*n* = 556)	*p*
Eating behavior	Optimal pattern	9 (19%)	59 (31%)	88 (30%)	156 (29%)	0.555^2^
	Normal pattern	16 (33%)	54 (28%)	87 (30%)	157 (30%)	
	Unfavorable pattern	23 (48%)	78 (41%)	117 (40%)	218 (41%)	
	n missing	2	8	15	25	
Fast-food consumption	> 1 serving per week	11 (22%)	30 (15%)	68 (23%)	109 (20%)	0.132^2^
	< = 1 serving per week	38 (78%)	165 (85%)	233 (77%)	436 (80%)	
	n missing	1	4	6	11	
Sporting activity	< 2 h per week	41 (82%)	141 (71%)	216 (70%)	398 (72%)	0.229^2^
	> = 2 h per week	9 (18%)	58 (29%)	91 (30%)	158 (28%)	
	n missing	0	0	0	0	
Cigarette use	yes	17 (34%)	83 (42%)	138 (55%)	238 (43%)	0.323^2^
	no	33 (66%)	116 (58%)	169 (45%)	318 (57%)	
	n missing	0	0	0	0	
Alcohol consumption	Risky alcohol consumption	9 (18%)	58 (29%)	103 (34%)	170 (31%)	0.074^2^
	Unrisky alcohol consumption	41 (82%)	141 (71%)	204 (66%)	386 (69%)	
	n missing	0	0	0	0	
BMI	X (SD)	24.7 (6.0)	24.4 (4.5)	25.3 (5.8)	24.9 (5.4)	0.171^1^
	n missing	1	4	13	18	
Self-rated health status	X (SD)	3.0 (0.8)	3.4 (0.8)	3.6 (0.8)	3.4 (0.8)	<0.001^1^
	n missing	0	0	0	0	
Psychological well-being	X (SD)	12.5 (6.0)	13.6 (5.2)	14.7 (5.2)	14.1 (5.3)	0.008^1^
	n missing	0	0	0	0	
Psychological well-being (categorized)	High psychological well-being	28 (56%)	124 (62%)	201 (66%)	353 (64%)	0.397^2^
	Low psychological well-being	22 (44%)	75 (38%)	106 (35%)	203 (37%)	
	n missing	0	0	0	0	
Medically diagnosed diseases	None	25 (50%)	101 (51%)	124 (40%)	250 (45%)	0.133^2^
	1–2 diseases	16 (32%)	74 (37%)	137 (45%)	227 (41%)	
	3–4 diseases	9 (18%)	21 (11%)	39 (13%)	69 (12%)	
	5 or more diseases	0 (0%)	2 (1%)	7 (3%)	9 (2%)	
	n missing	0	1	0	1	

1 ANOVA.

2 Pearson’s chi-squared test.

### 3.5 Associations between health literacy, self-efficacy and health behavior

The study found no statistically significant associations between health literacy and total health behavior score (Beta = 0.03, *p* = 0.541) or between self-efficacy and total health behavior score (*p* = 0.587). The covariates in this analysis were age, gender and education level.

Looking into the individual components of the health behavior score, the following results were obtained:

The study found no statistically significant associations between health literacy and smoking (*p* = 0.530), physical activity levels (*p* = 0.490), alcohol consumption (*p* = 0.608), nutritional patterns (*p* = 0.833) or fast-food consumption (*p* = 0.167).

Furthermore, the study did not establish a statistically significant association between self-efficacy and smoking (*p* = 0.749), physical activity levels (*p* = 0.695), alcohol consumption (*p* = 0.090), nutritional patterns (*p* = 0.139) or fast-food consumption (*p* = 0.264).

Since no statistically significant associations were observed between health literacy, self-efficacy and health behavior factors, the criteria for a mediation analysis have not been met.

### 3.6 Associations between health literacy, self-efficacy and health-related outcomes

Table 4 shows the associations between the variables of interest. Significant associations were found between all predictors and outcome variables. As a result, the criteria for mediation analyses have been met.

**Table 4:** Descriptive statistics and correlations between the main study variables.

Correlations							
		M	SD	1	2	3	4
1	Self-efficacy	28.97	4.43	–			
2	Health literacy	12.44	2.76	0.31**	–		
3	Psychological well-being	14.09	5.33	0.32**	0.15**	–	
4	Self-rated health status	3.44	0.81	0.32**	0.19**	0.43**	–

*p < 0.05; **p < 0.01.

### 3.7 Mediation analyses

#### 3.7.1 Associations between health literacy, self-efficacy and self-rated health

**Figure 1:**
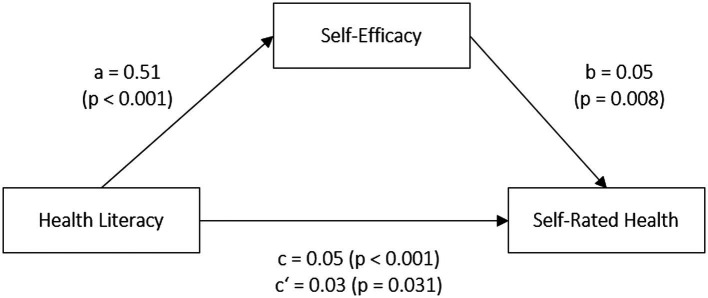
Mediation model: Self-efficacy as a mediator in the relationship between health literacy and self-rated health status.

Figure 1 shows the results of the mediation analyses with self-rated health as the dependent variable. The covariates in the mediation model were age, gender and education level. Path a, the effect of health literacy (independent variable) on self-efficacy (mediator variable) was statistically significant (*ß* = 0.51, *t* = 7.83, *p* < 0.001). There was a direct positive association between self-efficacy and self-rated health status (path b: *ß* = 0.05, *t* = 6.60, *p* = 0.008). The total effect of health literacy on self-rated health (path c: *ß* = 0.05, *t* = 4.35, *p* < 0.001) is partly explained by self-efficacy. A Sobel test confirmed the significant indirect effect of health literacy on self-rated health via self-efficacy (*Z* = 5.06, *p* < 0.001).

#### 3.7.2 Associations between health literacy, self-efficacy and psychological well-being

**Figure 2:**
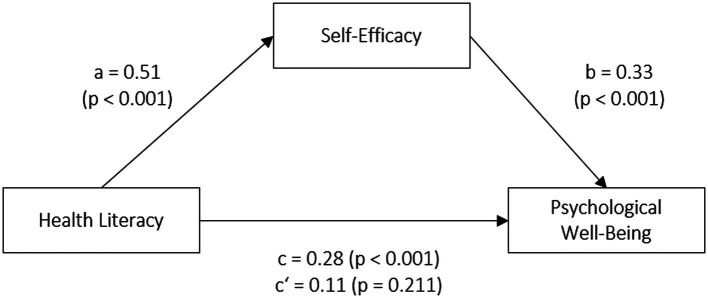
Mediation model: Self-efficacy as a mediator in the relationship between health literacy and psychological well-being.

Figure 2 shows the results of the mediation analyses with psychological well-being as the dependent variable. The covariates in the mediation model were age, gender and education level. Once again, health literacy was significantly associated with self-efficacy. There was a positive association between self-efficacy and psychological well-being (path b: *ß* = 0.33, *t* = 6.30, *p* < 0.001). The total effect of health literacy on psychological well-being (path c: *ß* = 0.28, *t* = 3.34, *p* < 0.001) is fully explained by self-efficacy. A Sobel test confirmed the significant indirect effect of health literacy on psychological well-being via self-efficacy (*Z* = 4.91, *p* < 0.001).

## 4 Discussion

In this study, we were able to investigate the relationships between health literacy, self-rated health status and psychological well-being, in addition to the mediating role of self-efficacy in a cohort of trainees and students in nursing in northern Germany at the beginning of their vocational training.

### 4.1 Health literacy and demographic traits

The findings of this study indicate that nearly every second participant had a limited level of health literacy. This is consistent with existing literature (10, 12, 18). However, a representative German study found the incidence of limited health literacy to be higher at above 60% for young adults aged 18–29 (11). In contrast to other studies (10–12), there were no statistically significant differences in health literacy scores between different age groups. However, this might be due to the rather homogeneous age distribution in the present cohort which had a preponderance of younger participants, and is in agreement with a comparable study on trainees (14). In contrast to the literature (10–12, 18) men showed statistically significantly higher levels of health literacy compared to women in the study cohort. However, these differences were only 0.6 on average on a scale ranging from 0 to 16. It should also be noted that men made up just 23% of the participants in this study, which might make it difficult to draw gender-specific conclusions. The gender distribution in our study, however, reflects the high proportion of women working in nursing (42).

Participants of German nationality were found to have significantly higher health literacy scores than participants of other nationalities, which is in concordance with a representative German study (11). There were no statistically significant differences in HL scores between trainees and students.

### 4.2 Health literacy, health behavior and health status

The prevalence of smoking (43%) was slightly higher in the cohort compared with data on a similar age group (40%) (43). 72% of the study population were found to have a low level of sporting activity; 44% reported less than 1 h/week, which is below WHO recommendations for physical activity (44). No significant associations between health literacy and health behavior could be observed in the cohort. This is in contrast to a systematic review performed by Fleary and colleagues, which found that 13 of the 17 included studies reported significant associations between health literacy and different aspects of health behavior in adolescents (45). A recent meta-analysis found small but significant effects of health literacy on health behavior (46). However, there is still a level of inconsistency in many studies investigating these relationships. A representative Danish study found that health literacy was strongly associated with physical activity, but not with smoking, and only to a small extent with alcohol consumption (12). Similarly, Olisarova et al. observed significant associations between health literacy and physical activity, but not smoking or alcohol consumption (47). However, a different study found that smokers with a higher level of health literacy were more likely to want to quit smoking than smokers with lower health literacy (48). A recent study on healthcare professionals found significant associations between health literacy and a health behavior score but did not examine associations with the individual components of the score (49). Overall, health literacy may be more likely to be associated with certain aspects of health behavior. However, it is worth noting that studies vary greatly in how they measure health literacy and health behavior outcomes. In general, measuring health behavior, a construct that is overly complex, dynamic and might change and vary frequently, is a challenging task for research and might lead to the inconsistencies we see in many studies. Furthermore, a significant share of the research in this field focuses on target groups with certain disease patterns rather than young and healthy populations, such as the cohort in this study. Concerning the lack of significant associations between health literacy and health behavior in this study, it is possible that the chosen variables and their operationalization do not accurately reflect the important aspects of young adult’s health behavior.

Studies are more consistent when it comes to the positive associations between health literacy and indicators of health status (12, 14, 46, 50–52). Our results align with these studies, indicating significant associations between health literacy, self-rated health status and psychological well-being. Having said this, our findings did not indicate any significant association between health literacy and BMI or the number of medically diagnosed diseases in this cohort.

### 4.3 Self-efficacy, demographic traits, health behavior and health status

The average self-efficacy level in the study cohort was around 29 points, with men having significantly higher scores. Other studies on healthy young adults reported average self-efficacy scores between 28 and 30 points, with contradictory results regarding gender differences (53–57). Higher age groups were significantly associated with higher self-efficacy scores, in contrast to other studies (54, 58). However, comparing this finding with those of other studies is challenging due to the narrow age range in the sample. Self-efficacy levels did not differ between trainees and students to a statistically significantly extent in this sample. We did not observe significant associations between self-efficacy and health behavior aspects. Although numerous studies have found associations between self-efficacy and indicators of health behavior (46, 57–60), the effects were mostly small and no consistent relationship could be observed.

Self-efficacy was, however, significantly associated with self-rated health status and psychological well-being in the cohort, which has also been demonstrated in other studies (60, 61). Nursing students and trainees with higher levels of self-efficacy were more likely to demonstrate better self-rated health status and psychological well-being.

### 4.4 Mediation analyses

The results of our analyses support previous findings that self-efficacy mediates the relationships between health literacy and both physical and mental health status. Kim and Yu observed a mediating role of self-efficacy in the effect of health literacy on physical and mental health status in older Korean adults living in community dwellings (21). Two studies found that self-efficacy mediated the relationship between health literacy and quality of life in patients with coronary heart disease (62) and tuberculosis (25). Furthermore, Stock et al. found that self-efficacy acted as mediator between health literacy and the number of chronic diseases (28). To our knowledge, this is the first study to investigate a group of nursing trainees and students in the very first year of their vocational training and analyze the relationships between health literacy, self-efficacy, health behavior and health status. In this cohort, self-efficacy partially mediated the relationship between health literacy and self-rated health, and fully mediated the relationship between health literacy and psychological well-being. This aligns with previous research performed in other target groups. In this regard, Sheeran et al. found in their meta-analysis that interventions were successful in increasing self-efficacy with a moderate overall effect size (59), indicating that self-efficacy is in fact a modifiable resource. Consequently, it is crucial to consider and target self-efficacy when designing interventions and studies targeting health literacy.

## 5 Limitations

There are some limitations to this study. Because this study is a cross-sectional study, causal relationships cannot be inferred from the correlations between variables. Furthermore, in cross-sectional research, it is not possible to determine cause and effect since all data are collected at one time point. It can also not determine whether one variable causes change in another or capture processes and developments. A longitudinal design should be aspired to in the future. As a result, four more follow-up assessments will be performed for this cohort at intervals of one year in line with our study protocol. Our results are based on first-year nursing trainees and students from northern Germany only and may not be applicable to other regions and populations. Participation was voluntary, which may lead to participants who are aware of health literacy or self-efficacy being over-represented. The low response rate is prone to selection bias and further limits the degree of generalizability, because participants in our study might differ systematically from the non-responders. This point is particularly important for the group of trainees, rather than for students, because the response rate in this study was especially low among trainees. The manner in which questionnaires were disseminated differed between trainees and students. Students were approached in person by the researchers at university lectures and time was provided during the lecture to complete the questionnaires. The questionnaires for the trainees were disseminated by the cooperating vocational schools. The exact conditions under which trainees were recruited are therefore unclear to the researchers and might even differ between vocational schools. The differences between these procedures are a probable explanation for the profound differences in the response rates of trainees and students. It is also worth noting that this study used paper-pencil questionnaires in the recruitment of trainees, which might have been disadvantageous in such a young cohort. Future research in young adults should rather focus on digital questionnaires and recruitment strategies.

Furthermore, all data was self-reported. As we investigated a rather young cohort, self-reported data may be flawed due to the small amount of health-related experience of young adults. This might be a particularly significant issue when it comes to self-rated health literacy (63). Moreover, a qualitative study found that adolescents had problems understanding items of the German long version of the HLS-EU questionnaire (64), which might also be applicable to the short version used in this study. Further research is needed to clarify the applicability of self-assessed health literacy measures in adolescents and young adults, for example by comparing self-assessment and objective measurements. Additionally, the study took place during the COVID-19 pandemic, which potentially influenced participants’ perception of their health status. Finally, the imbalanced gender ratio in the investigated cohort is a limitation worth noting. However, it is also reflective of the target population.

## 6 Conclusion

This study is the first to demonstrate the mediating effect of self-efficacy in the relationship between health literacy and physical and mental health status in first-year nursing students and trainees. The positive association between health literacy and physical health was partially mediated by self-efficacy, while self-efficacy fully mediated the association between health literacy and mental health status. The role of health behavior remains unclear since no significant associations were observed in this cohort. Reinforcing the health literacy and self-efficacy of students and trainees by incorporating suitable learning modules into the curriculum might improve health outcomes and contribute to a healthy nursing workforce.

## Data Availability

The raw data supporting the conclusions of this article will be made available by the authors, without undue reservation.
